# Old Drug, New Trick: Tilorone, a Broad-Spectrum Antiviral Drug as a Potential Anti-Fibrotic Therapeutic for the Diseased Heart

**DOI:** 10.3390/ph14030263

**Published:** 2021-03-15

**Authors:** Duncan Horlock, David M. Kaye, Catherine E. Winbanks, Xiao-Ming Gao, Helen Kiriazis, Daniel G. Donner, Paul Gregorevic, Julie R. McMullen, Bianca C. Bernardo

**Affiliations:** 1 Baker Heart and Diabetes Institute, Melbourne, VIC 3004, Australia; duncan.horlock@baker.edu.au (D.H.); david.Kaye@baker.edu.au (D.M.K.); catherinewinbanks@gmail.com (C.E.W.); xiaoming.gao@baker.edu.au (X.-M.G.); helen.kiriazis@baker.edu.au (H.K.); daniel.donner@baker.edu.au (D.G.D.); pgre@unimelb.edu.au (P.G.); julie.mcmullen@baker.edu.au (J.R.M.); 2 Department of Cardiology, Alfred Hospital, Melbourne, VIC 3004, Australia; 3 Department of Medicine, Monash University, Clayton, VIC 3800, Australia; 4 Baker Department of Cardiometabolic Health, University of Melbourne, Parkville, VIC 3010, Australia; 5 State Key Laboratory of Pathogenesis, Prevention and Treatment of High Incidence Disease in Central Asia, Clinical Medical Research Institute of Xinjiang Medical University, Urumqi 830054, China; 6 Centre for Muscle Research, Department of Physiology, University of Melbourne, Parkville, VIC 3010, Australia; 7 Department of Diabetes, Central Clinical School, Monash University, Clayton, VIC 3800, Australia; 8 Department of Physiology, Monash University, Clayton, VIC 3800, Australia; 9 Department of Physiology, Anatomy and Microbiology, La Trobe University, Melbourne, VIC 3086, Australia; 10 Department of Paediatrics, University of Melbourne, VIC 3010, Australia

**Keywords:** heart failure, fibrosis, tilorone, pressure overload, fibroblast, treatment, therapy

## Abstract

Cardiac fibrosis is associated with most forms of cardiovascular disease. No reliable therapies targeting cardiac fibrosis are available, thus identifying novel drugs that can resolve or prevent fibrosis is needed. Tilorone, an antiviral agent, can prevent fibrosis in a mouse model of lung disease. We investigated the anti-fibrotic effects of tilorone in human cardiac fibroblasts in vitro by performing a radioisotopic assay for [^3^H]-proline incorporation as a proxy for collagen synthesis. Exploratory studies in human cardiac fibroblasts treated with tilorone (10 µM) showed a significant reduction in transforming growth factor-β induced collagen synthesis compared to untreated fibroblasts. To determine if this finding could be recapitulated in vivo, mice with established pathological remodelling due to four weeks of transverse aortic constriction (TAC) were administered tilorone (50 mg/kg, i.p) or saline every third day for eight weeks. Treatment with tilorone was associated with attenuation of fibrosis (assessed by Masson’s trichrome stain), a favourable cardiac gene expression profile and no further deterioration of cardiac systolic function determined by echocardiography compared to saline treated TAC mice. These data demonstrate that tilorone has anti-fibrotic actions in human cardiac fibroblasts and the adult mouse heart, and represents a potential novel therapy to treat fibrosis associated with heart failure.

## 1. Introduction

Heart failure (HF), the end result of a number of cardiovascular disease states, is a life-threatening disorder affecting at least 26 million people worldwide, markedly affecting patients’ quality of life [1]. Caring for patients with HF comes at a significant economic cost, posing a burden not only on healthcare systems, but also placing significant emotional, physical, and financial costs for caregivers. Despite improvements in care, cardiovascular medicine, and surgery, the outlook for patients with HF remains poor, with almost 50% of patients with HF dying within 5 years of first diagnosis [2,3]. As a multifactorial clinical syndrome, HF remains a challenge to treat. New and effective therapies are needed for patients with types of HF for which current treatments (e.g., angiotensin converting enzyme inhibitors, beta blockers, diuretics) largely relieve symptoms rather than treating the disease [4,5,6,7,8]. Although these current pharmacological agents can enable patients to live longer, decrease hospital admissions, and are generally well tolerated, it is not uncommon for patients to experience adverse side effects (reviewed in [9]). Furthermore, more affordable therapies are urgently required, especially for those in developing nations. 

A key mechanism of HF is pathological cardiac remodelling, including cardiomyocyte hypertrophy, apoptosis, autophagy and myocardial fibrosis, leading to impaired left-ventricular (LV) function [10]. Cardiac fibrosis is associated with various forms of HF, and is usually classified as either replacement (associated with myocardial infarction, hypertrophic cardiomyopathy) or interstitial (usually occurs in aging and hypertension) fibrosis [11]. The deposition of excessive extracellular matrix proteins by cardiac fibroblasts has a significant effect on reducing heart chamber compliance, impairing normal heart function. This ultimately leads to adverse cardiac events. Despite its prevalence, there are no effective therapies in the clinic for inhibiting or reversing cardiac fibrosis. Furthermore, available therapies for HF are ineffective at preventing the formation of further fibrosis, highlighting the importance of developing novel and efficacious therapies that can resolve or prevent cardiac fibrosis.

Tilorone dihydrochloride is a small molecule that is orally bioavailable. It is currently used clinically as an antiviral agent in several countries outside of the USA. With recent virus outbreaks (Ebola and the Zika virus) and the current global pandemic caused by the novel coronavirus 2 (SARS-CoV-2) that emerged in 2019, there is renewed interest in tilorone’s antiviral mechanism and additional activities [12,13]. A high-throughput drug screen identified tilorone as a bone morphogenetic protein (BMP)-inducing agent, and was able to show sustained effects on gene expression of BMP7 and its target, inhibitor of differentiation-3 in vitro [14]. Its potential as an anti-fibrotic agent was first shown in a mouse model of pulmonary fibrosis, where in vivo administration of tilorone was able to significantly reduce the fibrotic response, and decrease the expression of extracellular matrix proteins collagen 1 and collagen 3 [14]. As BMP7 signalling is dysregulated in mouse models of HF and in patients with aortic stenosis [15], and administration of recombinant BMP7 (rh-BMP7) protein had beneficial effects in the prevention of fibrosis in mouse models of HF [15,16], we investigated the anti-fibrotic effects of tilorone in human cardiac fibroblasts in vitro and in vivo using a mouse model of pathological cardiac remodelling due to pressure overload. The identification of an alternative use for tilorone, a drug that is cheap to manufacture and has a history of clinical use [12], warrants further investigation, as such a drug could yield substantial therapeutic benefit in conditions where BMP7 signalling is dysregulated and fibrosis is a key feature.

## 2. Results

### 2.1. Tilorone Attenuates Transforming Growth Factor β (TGFβ) Induced Collagen Synthesis In Vitro

**Figure 1:**
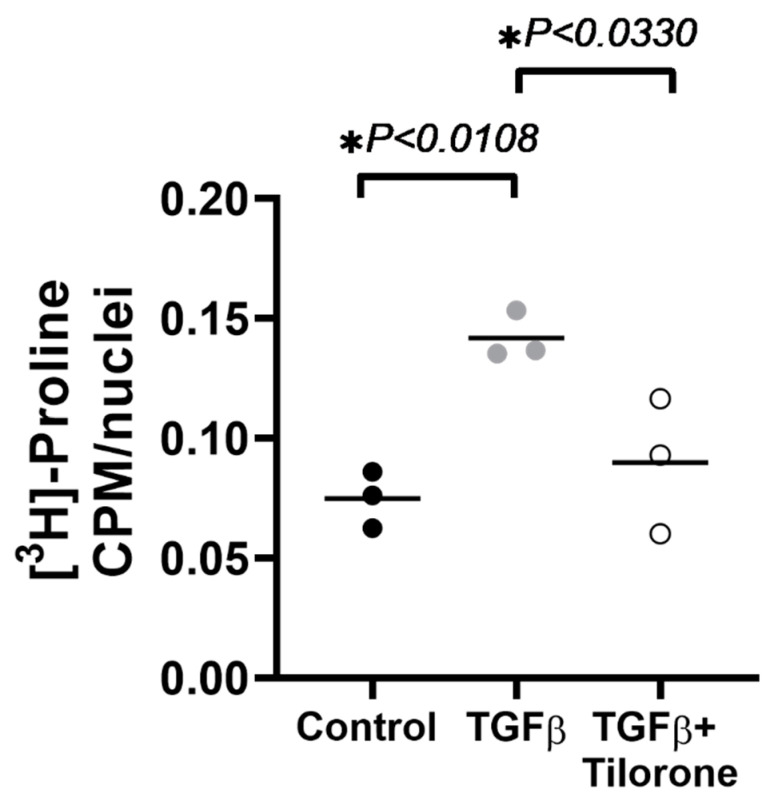
Anti-fibrotic effects of tilorone in human left ventricular cardiac fibroblasts in vitro. Tilorone attenuates TGFβ stimulated collagen synthesis in human left ventricular cardiac fibroblasts. Data analysed using a one-way ANOVA with Tukey’s post hoc test. Data passed Shapiro–Wilk test for normality. *n* = 3 independent experiments (average of 6 technical replicates per experiment). Lines indicate the mean. * p < 0.05.

We first determined whether tilorone had anti-fibrotic actions in a clinically relevant setting in vitro. The effect of tilorone on TGFβ induced collagen biosynthesis in human cardiac fibroblasts was determined by a [^3^H]-proline radioisotopic assay. In a pilot study, we first confirmed that tilorone had no effect on non-stimulated fibroblasts compared to control fibroblasts (Figure S1). In a follow up preliminary study, human cardiac fibroblasts expressed increased levels of collagenous proteins after stimulation with TGFβ by ~2-fold when compared to untreated fibroblasts (Figure 1). In contrast, human cardiac fibroblasts treated with tilorone showed markedly lower TGFβ induced collagen synthesis (~37% decrease vs. TGFβ treated fibroblasts). This was also not significantly different from control (i.e., untreated/unstimulated) fibroblasts (Figure 1). 

### 2.2. Tilorone Attenuates Cardiac Fibrosis in a Mouse Model with Pre-Existing Pathological Cardiac Remodelling and Cardiac Dysfunction due to Pressure Overload

**Figure 2:**
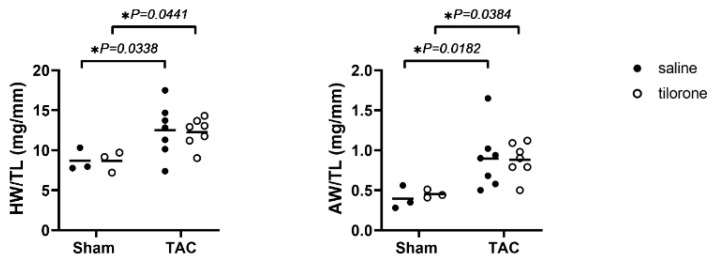
Tilorone does not attenuate TAC-induced cardiac hypertrophy. Heart weight (HW) and atria weight (AW) normalised to tibial length (TL). Data analysed using a two-way ANOVA with Fisher’s post hoc test. *n* = 3 (sham groups), 7 (TAC groups). Lines indicate the mean. * p < 0.05.

To determine whether tilorone could attenuate cardiac fibrosis in vivo, we used a pressure overload mouse model induced by transverse aortic constriction (TAC). This model recapitulates several characteristics of human HF (e.g., heart enlargement, impaired cardiac function, increased fibrosis). Of clinical relevance, TAC mimics increased left ventricular (LV) afterload in patients, which in the clinical setting predominantly occurs owing to aortic stenosis or systemic hypertension [17]. Mice with established pathological remodelling due to four weeks of TAC were administered tilorone (50 mg/kg, intraperitoneal, (i.p)) or saline every third day for eight weeks. Pathological cardiac hypertrophy together with depressed cardiac function is typically associated with an increase in heart and atrial size, and lung congestion [18,19]. Both TAC saline and TAC tilorone treated mice developed significant hypertrophy (~40% increase in heart weight (HW)/tibial length (TL) ratio vs. sham mice), and atrial enlargement (~95–125% increase in atria weight (AW)/TL ratio vs. sham mice) (Figure 2, Table 1), with negligible differences between tilorone and saline TAC groups. This is not unexpected as rh-BMP7 treatment in a previous study had no effect on cardiomyocyte cell size in TAC mice compared to vehicle treated TAC mice [16].

**Figure 3:**
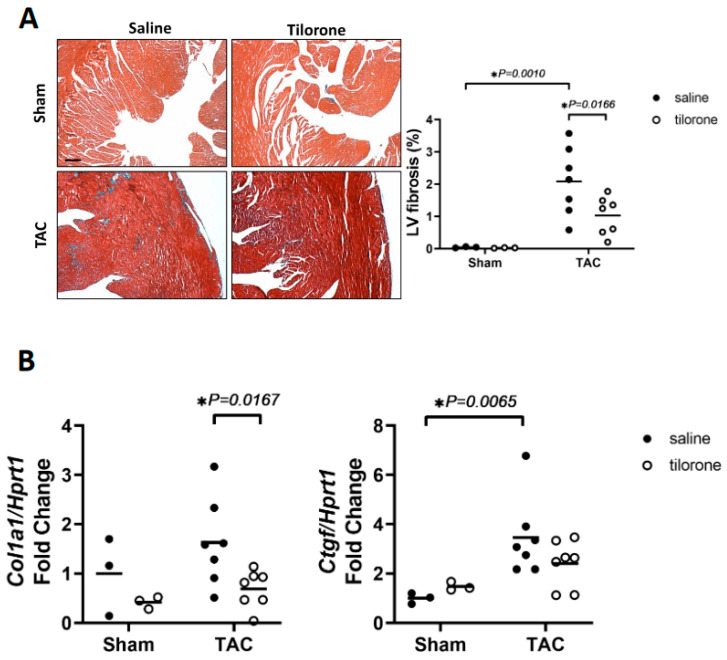
Tilorone attenuates TAC-induced fibrosis and is associated with a favourable fibrotic gene expression profile. (**A**) Representative left ventricular cross-sections stained with Masson’s trichrome from sham and TAC male mice treated with either saline or tilorone, and quantification of fibrosis. Scale bar = 200 μM. Data analysed by a one-way ANOVA with Fisher’s post hoc test. Lines represent the mean. *n* = 3 (sham groups), 7 (TAC groups). (**B**) qPCR quantification of Col1a1 and Ctgf gene expression relative to Hprt1 in Sham and TAC saline and tilorone treated hearts. Data analysed using a two-way ANOVA with Fisher’s post hoc test. Lines represent the mean. *n* = 3 (Sham groups), 7 (TAC groups). * *p <* 0.05.

Analysis of Masson’s trichrome-stained sections of hearts from mice that had received tilorone showed a significant attenuation of fibrosis within the LV by about 50% as compared to saline-treated TAC mice (Figure 3A). The increase in fibrosis following aortic banding is typically associated with increased levels of profibrotic factors and extracellular matrix proteins [20]. The increased deposition of fibrosis in TAC saline mice was accompanied by increased gene expression of the matricellular protein connective tissue growth factor (Ctgf) which has a role in extracellular matrix deposition during developmental and pathological conditions [21,22] and was not significantly elevated in TAC tilorone-treated mice (Figure 3B). In addition, collagen 1 gene expression was attenuated with tilorone treatment in TAC mice compared to saline treated TAC control mice (Figure 3B). Collectively, our in vitro and in vivo data suggest that tilorone has anti-fibrotic properties in the diseased heart.

### 2.3. Treatment with Tilorone Is Associated with a Favourable Cardiac Stress Gene Expression Profile

**Figure 4:**
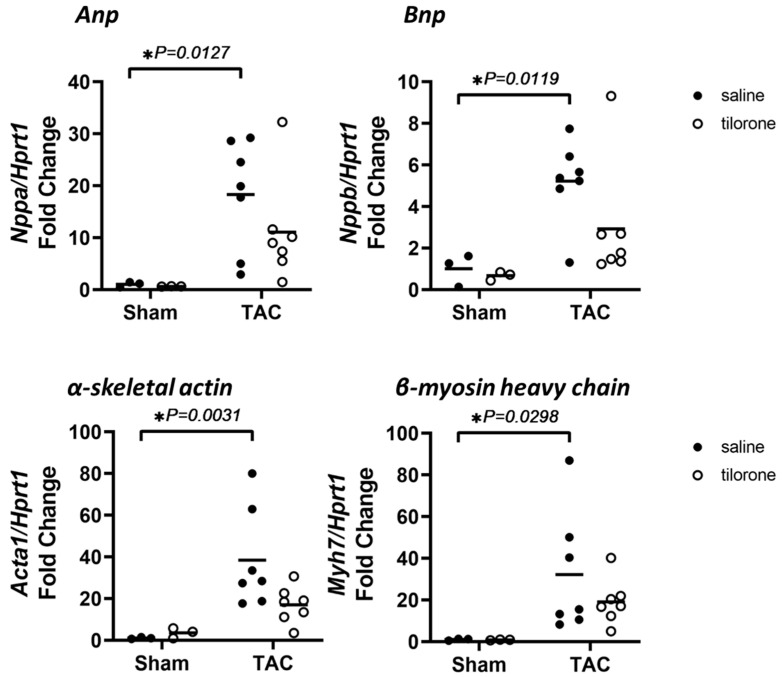
Treatment with tilorone is associated with a favourable cardiac stress gene expression profile. qPCR quantification of *Nppa (Anp), Nppb (Bnp), Acta1 (α skeletal actin)* and *Myh7 (β-MHC)* gene expression relative to *Hprt1* in Sham and TAC saline and Tilorone treated hearts. Data analysed using a two-way ANOVA with Fisher’s post hoc test. Lines represent the mean. *n* = 3 (sham groups), 7 (TAC groups). * *p <* 0.05.

Pressure overload-induced pathological hypertrophy is associated with the re-expression of the cardiac foetal gene program [10]. The cardiac foetal genes atrial natriuretic peptide (ANP), B-type natriuretic peptide (BNP), α skeletal actin and β-myosin heavy chain (βMHC) were all significantly elevated in TAC saline treated mice but not in TAC tilorone treated mice (Figure 4).

### 2.4. Treatment with Tilorone Prevented a Further Decline in Cardiac Function

**Figure 5:**
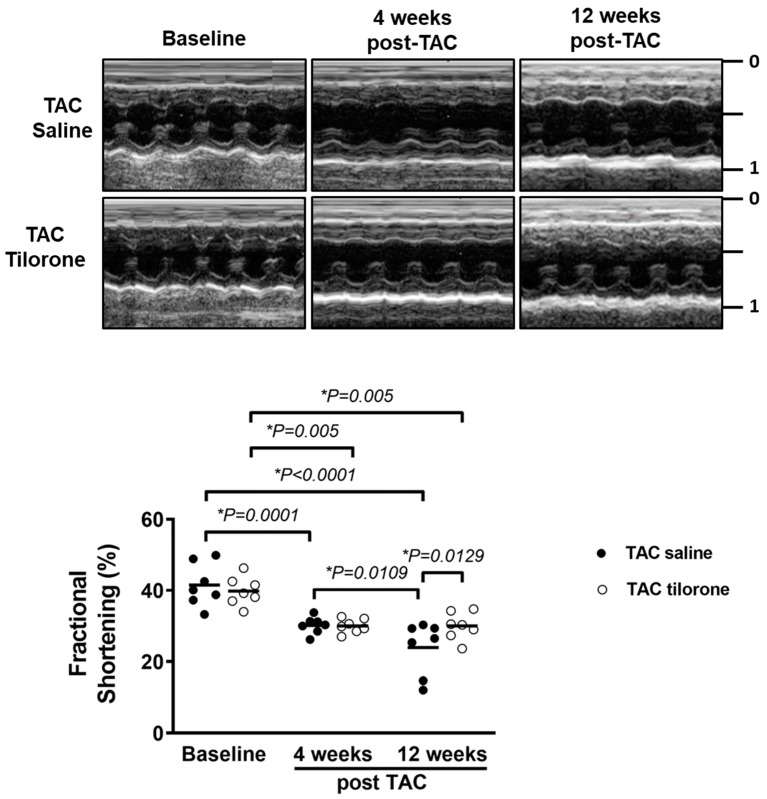
Treatment with tilorone prevented a further decline in cardiac function. Top: Representative M-modes at baseline, 4 weeks post-TAC and endpoint. Bottom: Quantification of fractional shortening at baseline (pre-surgery), 4 weeks post-TAC (prior to saline/tilorone administration), and 8 weeks post-treatment (i.e., 12 weeks post-TAC). Saline/tilorone treatment commenced at 4 weeks post-TAC. Data analysed using a two-way Repeated Measures ANOVA with Fisher’s post hoc test. Lines represent the mean. *n* = 7 (TAC groups). * *p <* 0.05.

Following 4 weeks of pressure overload, TAC mice displayed increased LV wall thickness and depressed cardiac function (indicated by a decrease of 25–30% in fractional shortening [FS], which is a measure of systolic function, 4 weeks post TAC compared with pre-surgery values; Figure 5, Table 2). Following 8 weeks of treatment, cardiac function in TAC saline mice further deteriorated (FS decreased by a further 18% at endpoint compared to 4 weeks post-TAC; Figure 5, Table 2). In contrast, treatment with tilorone prevented a further decrease in cardiac function in TAC tilorone mice as FS remained unchanged between 4 weeks post-TAC and after 8 weeks of treatment (Figure 5, Table 2). 

## 3. Discussion

Cardiac fibrosis, due to enhanced/inappropriate deposition of extracellular matrix proteins in cardiac tissue, is a process whereby heart tissue is progressively replaced with scar tissue leading to stiffening of the heart and impaired heart function and eventually HF [23]. Importantly, fibrosis is a common feature of advanced HF independent of the aetiology of the cardiomyopathy. Whilst new tools are available to assess cardiac fibrosis in clinical trials, (e.g., MRI [24]), and available drugs such as anti-hypertensive therapies and anti-inflammatory agents are under investigation [25], there are currently no approved therapies that can specifically treat HF-associated fibrosis. In previous studies, a role for the BMP pathway in HF-induced fibrosis is supported by the ability to reduce collagen deposition and improve cardiac function in a mouse model of pressure overload [15,16,26]. However, in all three studies, BMP (delivered as either rh-BMP7 or BMP-2 via osmotic mini pumps) was administered at the time of surgery, before any LV hypertrophy, remodelling, and cardiac dysfunction had occurred. This is in contrast to our study, which delivered tilorone, a BMP-inducing drug [14], in a mouse model with established cardiac pathology and hypertrophy. To our knowledge, this is the first study to demonstrate the anti-fibrotic action of tilorone in the heart. To this end, we have been able to demonstrate that tilorone was able to: (i) attenuate TGFβ induced collagen synthesis in isolated human LV cardiac fibroblasts in a preliminary study; (ii) attenuate fibrosis in a mouse model of established pathological hypertrophy due to pressure overload, which is accompanied with a favourable cardiac gene expression profile; and (iii) prevent further decline in systolic cardiac function in vivo. Although tilorone did not have an anti-hypertrophic effect on the heart, this is not surprising, as rh-BMP7 treatment had no effect on cardiomyocyte cell size following pressure overload [16]. Whilst BMP7 recombinant protein has shown beneficial effects in the prevention of fibrosis in heart failure in preclinical mouse models [15,16], the cost of ongoing administration of recombinant BMP7 protein to HF patients is a major issue that hinders its progression for therapeutic application [27]. Key advantages of developing tilorone as a therapeutic for the treatment of HF are: (i) it can be administered orally, (ii) it is a small molecule and relatively cheap to manufacture, (iii) it is stable which impacts on storage, shelf life and transport, (iv) it is known to have a very long plasma residence time, so dosing and metabolic stability are not issues [12]. The value of tilorone as an anti-fibrotic therapy for cardiac disease deserves further attention which will elucidate whether tilorone could either be a stand-alone or adjunct therapy. Moving forward, the effect of tilorone can be tested in complementary mouse models that display features of fibrosis and cardiac dysfunction (e.g., myocardial infarction). Further, more detailed cardiac haemodynamic studies would be of interest to determine the effect of tilorone on diastolic function, where cardiac fibrosis is known to contribute to the pathogenesis of diastolic dysfunction [28] In addition, in vitro studies in human cardiac fibroblasts can be expanded to include a wide range of heart disease patient cohorts.

Finally, the therapeutic potential of tilorone could be two-fold: both as an anti-viral [12,13,29] and anti-fibrotic agent. In this current study, we have shown anti-fibrotic actions of tilorone in the heart. In a previous study, Leppäranta et al. demonstrated anti-fibrotic actions of tilorone in a mouse model of pulmonary fibrosis, where administration of tilorone decreased lung hydroxyproline content and the expression of collagen genes [14]. Taken together, these studies warrant further investigation of tilorone for more widespread testing against conditions where fibrosis is a key feature. Drugs that have a role in attenuating pro-fibrotic pathways, for example, could become particularly important for the treatment of complications following SARS-CoV-2 (COVID-19) infection. Emerging data suggests that the burden of fibrotic lung disease following COVID-19 infection is likely to be high [30,31], and myocardial injury present in >25% of severe cases [32]. Further, in recent studies in patients that have recovered from COVID-19 infection, cardiac magnetic resonance imaging has revealed cardiac involvement (myocardial oedema, fibrosis and function) in a proportion of patients [33,34].

## 4. Materials and Methods

### 4.1. Animal Experimental Procedures

All experiments using animals were conducted in accordance with the Australian code for the care and use of animals for scientific purposes (National Health and Medical Research Council of Australia, 8th Edition, 2013). All animal procedures and care were approved by the Alfred Research Alliance Animal Ethics Committee for Baker Heart and Diabetes Institute (approval number E/1426/2014/B, date of approval 20 February 2014). Male mice on a C57BL6 background were bred and housed onsite and maintained in a temperature-controlled environment under a 12 h light/dark cycle, with up to four littermates per cage. All mice had *ad libitum* access to standard rodent chow and water.

### 4.2. Study Design

**Figure 6:**
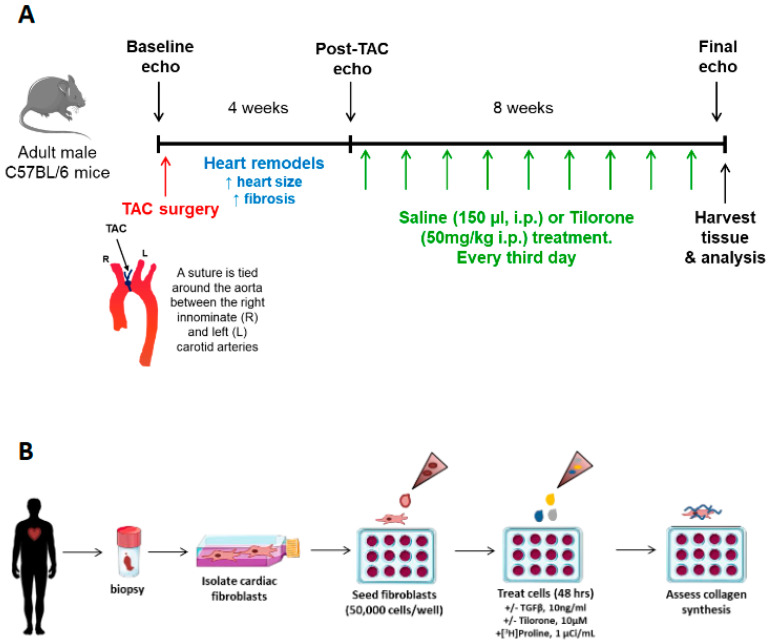
Experimental Outlines. (**A**) Timeline of animal study design. (**B**) Schematic demonstrating experimental workflow for the radioisotopic assay to determine collagen synthesis in vitro.

**Figure 7:**
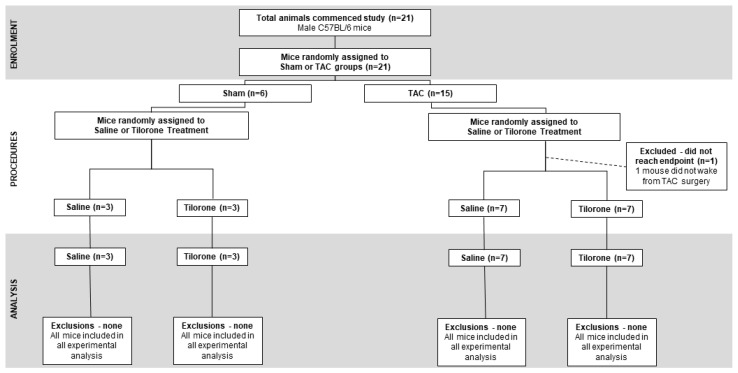
Experimental Animals and exclusions. A flowchart for the reporting of animal use, allocation, experimental analysis and exclusions following the CONsolidated Standards of Animal Experiment ReporTing (CONSAERT).

Following baseline echocardiography assessment, adult male mice (8 to 10 weeks old, *n* = 21) were subjected to pressure overload surgery induced by transverse aortic constriction (TAC, *n* = 15) or sham surgery (*n* = 6) and left for 4 weeks. During this period, the heart undergoes remodelling and is associated with an increase in heart size, fibrosis and decrease in cardiac function. Prior to treatment with tilorone and saline, heart remodelling in response to TAC was confirmed by echocardiography. Mice were randomly assigned into sham/TAC control or treated groups and dosed with saline (150 µL of equal volume, i.p., control group, *n* = 3 sham, *n* = 7 TAC) or Tilorone (50 mg/kg, i.p., treated group as per dosing schedule described in [14], *n* = 3 sham, *n* = 7 TAC) every third day for eight weeks. Echocardiography was performed 12 weeks post-TAC (i.e., 8 weeks post treatment) to determine the effects of tilorone treatment on cardiac function. Following the final echocardiography measurement, mice were euthanised, and tissue harvested for experimental analysis (Figure 6A). One TAC mouse was excluded from the study as it did not wake from surgery. A flowchart for the reporting of animal use, allocation, experimental analysis and exclusions following the CONsolidated Standards of Animal Experiment ReporTing (CONSAERT) guidelines as proposed by Weeks et al. [35] is provided in Figure 7.

### 4.3. Pressure Overload Induced by Transverse Aortic Constriction (TAC)

Male wildtype C57BL/6 mice were subjected to TAC or sham operation as previously described [18,19,36,37]. TAC is a surgical technique used to induce LV cardiac hypertrophy, allowing the study of novel therapeutic interventions for the treatment of cardiac hypertrophy and HF. The TAC model induces a chronic pressure load on the heart and is associated with progressive pathological hypertrophy and cardiac dysfunction within 4 weeks of surgery [38]. Mice were anaesthetised with ketamine, xylazine and atropine (100:20:1.2 mg/kg, i.p.), administered an analgesic (carprofen, 5 mg/kg, subcutaneously, (s.c.)) and intubated for ventilation during surgery. Mice were kept warm on a heat pad (~32 °C) in a supine position, administered a local anaesthetic (lignocaine, 7 mg/kg, s.c.) at the site of incision where a sternotomy was performed to access the aorta. A non-absorbable 5-0 braided silk suture was tied around the aorta between the right innominate and left carotid arteries, against a 26-gauge (0.46 mm) cannula. With removal of the cannula, the inner aortic diameter is reduced to about 0.4–0.5 mm. Following closure of the sternum, betadine antiseptic topical solution was spread over the wound, and mice were administered atipamezole (0.2 mg/kg, i.p., to help recovery from anaesthesia) and frusemide (4 mg/kg, i.p., diuretic; helps in reducing oedema post surgery) and monitored up to twice daily for a week post surgery. For sham operations to serve as controls, mice received the same surgical procedure except that the aorta was not banded. TAC surgery was performed by the same person to reduce variability.

### 4.4. Left Ventricular Structure and Function

To obtain measures of LV systolic function, transthoracic echocardiography (M-mode echocardiography) was performed on anaesthetised mice (1.8% isoflurane, inhalation) using a Philips iE33 ultrasound machine with a 15 MHz linear array transducer as previously described [18,19,36,37]. Echocardiography was performed prior to surgery (baseline), at 4 weeks post-surgery (and prior to treatment), and 8 weeks post-treatment (i.e., 12 weeks post-TAC). Briefly, anaesthetised mice were hand held in a supine position, acoustic coupling gel was placed on the shaved chest area, and images were acquired over an approximate 5 min recording. Image acquisition at each time point was performed by the same operator blinded to surgery and treatment. LV chamber dimensions (LV end-diastolic dimension, LVEDD; LV end-systolic dimension, LVESD), LV wall thickness at diastole (LV posterior wall, LVPW; Interventricular septum, IVS) were measured from short axis images of the LV. LV dimensions and wall thickness were measured from three beats per echocardiogram and averaged. Fractional shortening was calculated as follows: [(LVEDD − LVESD)/LVEDD] × 100%. Ejection fraction was calculated as follows: [(LVEDD^3^ − LVESD^3^)/LVEDD^3^] × 100%. Heart rate (HR) was measured from the time taken of ten beats (secs) per echocardiogram and converted to bpm. Body weights were obtained from conscious mice at the time of each echocardiography procedure. Data analysis was performed offline by the same person using dedicated software (ProSolv Cardiovascular Analyser, version 3.5; ProSolv, Indianapolis, IN, USA) who was blinded to treatment. All M-mode measurements were validated by an independent investigator following an internal quality control process [39].

### 4.5. Tissue Collection

At the study endpoint, mice were anaesthetised with a single i.p. injection of pentobarbitone (80 mg/kg) and when unconscious, euthanised by cervical dislocation and weighed to obtain final body weight. Tissues were quickly excised whole, rinsed in phosphate-buffered saline (137 mM NaCl, 2.7 mM KCl, 10 mM Na_2_HPO_4_, and 1.8 mM KH_2_PO_4_) to remove blood, patted dry on sterile gauze and weighed. The atria were then removed from the heart and weighed. All tissues were snap frozen in liquid nitrogen and stored at −80 °C for molecular analysis. Hind legs were removed, submerged in 1 M NaOH and incubated at 37 °C for 7–10 h to remove surrounding tissue. Tibias were cleaned and rinsed in distilled H_2_O and air dried. Tibia bone length was measured using a Vernier calliper.

### 4.6. Proline Assay

Patient cardiac fibroblasts were cultured from LV myocardial tissue obtained from explanted failing hearts at the time of heart transplantation (Figure 6B). Patients provided written informed consent, the study was approved by the Alfred Hospital Ethics and Research Committee, and it conformed to the principles outlined in Declaration of Helsinki. Isolated human cardiac fibroblasts were cultured with transforming growth factor-β (TGFβ, 10 ng/mL, which stimulates collagen production), in the presence or absence of tilorone (10 μM, dose described in [14]), and 1 μCi/mL [^3^H]-proline. After 48 h, cells were fixed and stained with Hoechst dye to count the number of nuclei prior to digestion in sodium hydroxide and addition to scintillation fluid. Scintillation counts were performed on a Hidex SL 600 Liquid Scintillation Counter as a measure of [^3^H]-proline incorporated into collagen. All radiation counts were normalised to nuclei number as a proxy for cell number. Each experiment consisted of six technical replicates for each treatment, and was repeated three independent times from the same cell line. 

### 4.7. RNA Isolation

Total RNA was isolated from frozen mouse ventricles using TRI Reagent (Sigma-Aldrich, St. Louis, MO, USA). Briefly, mouse ventricles were homogenised in 500 μL of TRI Reagent to disrupt cells to release and fragment cellular contents and then centrifuged to remove high molecular weight components, minimizing their presence in the aqueous phase. The supernatant containing RNA was collected, and a phenol-chloroform extraction is done to the Trizol homogenates by addition of 100 µL of chloroform and vortexed. To achieve phase separation, samples were centrifuged to separate aqueous and organic phases for 15 min at 4 °C. The upper aqueous phase containing RNA was recovered, and RNA extracted by isopropanol precipitation overnight at −20 °C. The precipitated RNA was pelleted by centrifugation for 15 min at 4 °C. The RNA pellets were washed twice with 75% ethanol, air dried and resuspended in 30–50 μL RNase-free water. RNA was quantified using the NanoDrop 1000 Spectrophotometer (Thermo Fisher, Wilmington, DE, USA). All RNA samples had a 260/280 ratio between 1.9–2.0 and a 260/230 ratio between 1.6−2.3. Purified RNA was stored at −80 °C.

### 4.8. Reverse Transcription

Total heart RNA (2 µg) was converted to cDNA using the using the High Capacity cDNA Reverse Transcription Kit (Thermo Fisher) in a 20 µL reverse transcription reaction containing the following components at a final concentration: 2X Reverse Transcription (RT) Buffer, 1X dNTP mix (4 mM), 1X RT Random Primers and 1 U/µL RNase Inhibitor. The RT protocol consisted of 10 min at 25 °C, 120 min at 37 °C, and 5 min at 85 °C in a thermo cycler. Following cDNA synthesis, heart cDNA was diluted to a final concentration of 25 ng/µL and stored at −20 °C.

### 4.9. Quantitative PCR (qPCR)

To measure cardiac mRNA expression, qPCR was performed on an Applied Biosystems 7500 real-time PCR instrument. mRNA expression was measured using the TaqMan^®^ Fast Advanced Master Mix and TaqMan^®^ Gene Expression Assays (20X primer/probe mix). The PCR conditions consisted of 20 s at 95 °C, then 40 cycles of 1 s at 95 °C, and 20 s at 60 °C. Hypoxanthine phosphoribosyltransferase 1 *(Hprt1)* was used to standardise for cDNA concentration and data were analysed using the 2^−ΔΔCt^ method of quantification.

### 4.10. Histological Analysis

The top half of heart samples were immediately fixed in 4% paraformaldehyde overnight at 4 °C following dissection to maintain tissue morphology. After fixation, heart tissue was dehydrated to enable embedding with paraffin (Alfred Hospital Pathology, Australia). Heart tissue was sectioned using a microtome at 6 μm cross-sections, dried and then rehydrated for staining with Masson’s trichrome stain to assess cardiac collagen deposition/interstitial fibrosis (Alfred Hospital Pathology). Images of the LV were captured using a light microscope at 40× magnification. To determine the percentage of collagen in the LV, the number of blue pixels (Masson’s trichrome stains collagen blue) and total area of the LV were counted using Image-Pro Analyzer 7.0 (Media Cybernetics Inc., Bethesda, MD, USA). The percentage of fibrosis in the LV was calculated by dividing the total amount of fibrosis in the LV by the total area of the LV and multiplying by 100%. All samples were analysed blind by the one person.

### 4.11. Statistical Analyses

Statistical analyses were performed using GraphPad Prism (Version 8.1.2, San Diego, CA, USA). The results in tables are presented as mean ± standard error of the mean (SEM). For cell culture studies, differences between groups were identified using a one-way analysis of variance (ANOVA) followed by Tukey’s post hoc test. For animal studies, differences between groups were identified using a two-way or one-way ANOVA followed by Fisher’s post hoc test. For echocardiography parameters, differences between groups were identified using a two-way repeated measures ANOVA followed by Fisher’s post hoc test. A value of *p* < 0.05 was considered significant. All relative units are expressed as a fold change with the relevant control group normalised to 1.

## 5. Conclusions

There are currently no approved therapies that can treat HF associated fibrosis. The development of new therapies is important, but the exploration of repurposing or optimisation of pre-existing drugs is also an important avenue of investigation. Here, we show tilorone, a 50 year old broad-spectrum antiviral drug, has biologically significant anti-fibrotic action in the heart that was previously unrealised. We demonstrated that tilorone can attenuate fibrosis and prevent cardiac functional decline in a mouse model with pre-existing pathological cardiac remodelling due to pressure overload, and can attenuate TGFβ-stimulated collagen synthesis in human cardiac fibroblasts. Our observations warrant further studies to determine whether tilorone could be a valuable anti-fibrotic therapy for the treatment of HF, either as a standalone therapy or combined with current mainstay therapies for HF. 

## Figures and Tables

**Table 1:** Morphological data at endpoint of sham and TAC saline- and tilorone-treated mice.

	Sham		TAC	
	Saline	Tilorone	Saline	Tilorone
No. of Animals	3	3	7	7
**Body Weight (g)**	30.6 ± 0.2	29.5 ± 0.6	30.2 ± 1.0	31.1 ± 0.7
**Tibial Length (mm)**	16.1 ± 0.1	16.1 ± 0.1	16.2 ± 0.1	16.3 ± 0.1
**Heart Weight (mg)**	140.2 ± 13.1	140.0 ± 11.8	203.3 ± 20.6	199.5 ± 10.5
**Atrial Weight (mg)**	6.4 ± 1.3	7.3 ± 0.5	14.6 ± 2.4 *	14.3 ± 1.3 *
**Lung Weight (mg)**	162.6 ± 7.6	176.3 ± 7.0	244.5 ± 55.0	203.4 ± 16.8
**HW/BW (mg/g)**	4.6 ± 0.5	4.7 ± 0.3	6.7 ± 0.7 *	6.4 ± 0.3
**AW/BW (mg/g)**	0.21 ± 0.05	0.25 ± 0.02	0.49 ± 0.09*	0.46 ± 0.04
**LW/BW (mg/g)**	5.3 ± 0.3	6.0 ± 0.1	8.3 ± 2.1	6.6 ± 0.6
**HW/TL (mg/mm)**	8.7 ± 0.8	8.7 ± 0.8	12.5 ± 1.2 *	12.3 ± 0.7 *
**AW/TL (mg/mm)**	0.40 ± 0.08	0.45 ± 0.03	0.90 ± 0.15 *	0.88 ± 0.08 *
**LW/TL (mg/mm)**	10.1 ± 0.5	10.9 ± 0.5	15.1 ± 3.4	12.5 ± 1.0

Abbreviations: AW, atria weight; BW, body weight; HW, heart weight; LW, lung weight; TL, tibial length. Data are shown as mean ± SEM. Data analysed by two way ANOVA with Fisher’s post hoc test. * *p* < 0.05 vs. Sham of same treatment.

**Table 2:** Echocardiography measurements of TAC saline and TAC Tilorone treated mice at baseline, 4 weeks post-TAC, and at endpoint (12 weeks post-TAC/8 weeks post-treatment).

	BASELINE		4 WEEKS POST-TAC		ENDPOINT	
	TAC		TAC		TAC	
	Saline	Tilorone	Saline	Tilorone	Saline	Tilorone
No. of Animals	7	7	7	7	7	7
**Body Weight (g)**	29.1 ± 0.4	28.9 ± 0.6	29.8 ± 0.6	29.8 ± 0.5	30.4 ± 1.2	30.9 ± 0.8
**Heart Rate (bpm)**	521 ± 16	559 ± 31	562 ± 14	546 ± 25	566 ± 22	574 ± 13
**LVPW (mm)**	0.80 ± 0.01	0.78 ± 0.02	1.11 ± 0.01 *	1.16 ± 0.01 *	1.07 ± 0.03 *	1.08 ± 0.02 *‡
**IVS (mm)**	0.79 ± 0.01	0.79 ± 0.01	1.15 ± 0.01 *	1.19 ± 0.01 *	1.08 ± 0.03 *	1.05 ± 0.04 *‡
**LVEDD (mm)**	3.89 ± 0.11	4.04 ± 0.06	4.24 ± 0.08 *	4.21 ± 0.10	4.32 ± 0.20 *	4.39 ± 0.12 *
**LVESD (mm)**	2.28 ± 0.14	2.43 ± 0.06	2.96 ± 0.07 *	2.95 ± 0.09 *	3.31 ± 0.28 *^#^	3.08 ± 0.14 *
**Fractional Shortening (%)**	42 ± 2	40 ± 2	30 ± 1 *	30 ± 1 *	24 ± 3 *‡	30 ± 1 *†
**Ejection Fraction (%)**	79 ± 2	78 ± 2	66 ± 1 *	66 ± 1 *	55 ± 5 *‡	65 ± 2 *†

Abbreviations: BW, body weight; HR, heart rate; LVPW, left ventricular posterior wall thickness; IVS, interventricular septum thickness; LVEDD, left ventricular end-diastolic dimension; LVESD, left ventricular end-systolic dimension; FS, fractional shortening. Data are shown as mean ± SEM. Data analysed using a two-way repeated measures ANOVA with Fisher’s post hoc test. * *p* < 0.05 vs. corresponding baseline group; ‡ *p* < 0.05 vs. 4 weeks of same group, † *p* < 0.05 vs. TAC saline at same timepoint, # *p* = 0.0529 vs. 4 weeks of same group.

## Data Availability

The data presented in this study is contained within the article and supplementary material.

## Supplementary Materials

The following are available online at https://www.mdpi.com/1424-8247/14/3/263/s1, Figure S1: Effect of tilorone in human cardiac fibroblasts.
