# Supplementary material for: Old Drug, New Trick: Tilorone, a Broad-Spectrum Antiviral Drug as a Potential Anti-Fibrotic Therapeutic for the Diseased Heart
Source: Pharmaceuticals (Basel). 2021 Mar 15;14(3):263. doi: 10.3390/ph14030263 (PMC7998193; doi:10.3390/ph14030263)
Supplement: Supplementary file 1 [file pharmaceuticals-14-00263-s001.pdf]

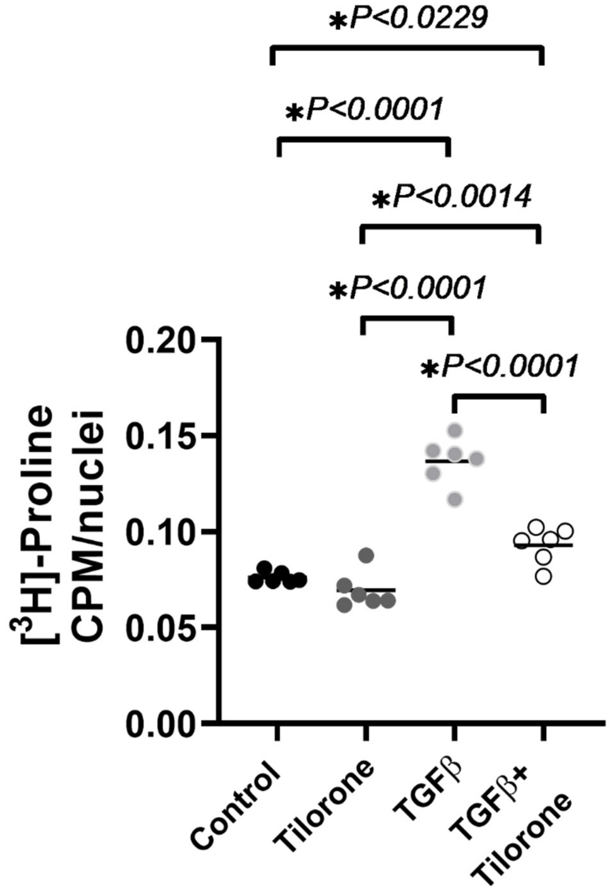

Figure 1. Effect of tilorone in human cardiac fibroblasts. Tilorone had no effect on non-stimulated fibroblasts compared to control fibroblasts. Tilorone attenuates TGFβ stimulated collagen synthesis in human left ventricular cardiac fibroblasts. Each data point represents technical replicates from one independent experiment. Data analysed using a one way ANOVA with Tukey's Post Hoc test. Lines indicate the mean. $^*P < 0.05$.
